# Study on the Regulatory Effects of GA_3_ on Soybean Internode Elongation

**DOI:** 10.3390/plants10081737

**Published:** 2021-08-23

**Authors:** Fuxin Shan, Rui Zhang, Jin Zhang, Chang Wang, Xiaochen Lyu, Tianyu Xin, Chao Yan, Shoukun Dong, Chunmei Ma, Zhenping Gong

**Affiliations:** 1 College of Agriculture, Northeast Agricultural University, Harbin 150030, China; sfx18846424314@163.com (F.S.); zhang134rui@163.com (R.Z.); 15263485352@163.com (J.Z.); 18249555621@163.com (C.W.); xiaochenlyu@163.com (X.L.); xty1289304581@163.com (T.X.); yanchao504@126.com (C.Y.); shoukundong@163.com (S.D.); chunmm518@163.com (C.M.); 2 College of Agronomy and Biotechnology, Yunnan Agricultural University, Kunming 650201, China

**Keywords:** soybean stem, gibberellic acid, griding, transportation

## Abstract

Excessive plant height is an important factor that can lead to lodging, which is closely related to soybean yield. Gibberellins are widely used as plant growth regulators in agricultural production. Gibberellic acid (GA_3_), one of the most effective active gibberellins, has been used to regulate plant height and increase yields. The mechanism through which GA_3_ regulates internode elongation has been extensively investigated. In 2019 and 2020, we applied GA_3_ to the stems, leaves, and roots of two soybean cultivars, Heinong 48 (a high-stalk cultivar) and Henong 60 (a dwarf cultivar), and GA_3_ was also applied to plants whose apical meristem was removed or to girded plants to compare the internode length and stem GA_3_ content of soybean plants under different treatments. These results suggested that the application of GA_3_ to the stems, leaves, and roots of soybean increased the internode length and GA_3_ content in the stems. Application of GA_3_ decreased the proportion of the pith in the soybean stems and primary xylem while increasing the proportion of secondary xylem. The apical meristem is an important site of GA_3_ synthesis in soybean stems and is involved in the regulation of stem elongation. GA_3_ was shown to be transported acropetally through the xylem and laterally between the xylem and phloem in soybean stems. We conclude that the GA_3_ level in stems is an important factor affecting internode elongation.

## 1. Introduction

Stem elongation stress is an important factor that can result in lodging of plants under shade stress [1]. Gibberellins (GAs) promote cell elongation and increase cell numbers and thus are a key factor involved in stem elongation [2,3,4,5]. Gibberellic acid (GA_3_) is a biologically active form of GAs [6]. When soybean (*Glycine max (Linn.) Merr.*) plants are subjected to shade stress, their internodes elongate while the stems become thinner, accompanied by increases in GA_3_ levels in the main stems and leaves [7,8]. Zhang et al. [9] reported that the increased GA_3_ content in the stems is the main reason for the change in internode length and diameter of soybean internodes in response to shading. According to Bawa et al. [10], the increase in GA_3_ and GA_7_ levels caused by the interaction of low light and high temperature promotes the elongation of soybean hypocotyls. Indeed, changes in the environment lead to an increase in stem GA_3_ levels and promote stem elongation, which has been confirmed in pea (*Pisum sativum* L.) [11], Himalayan lily (*Cardiocrinum giganteum*) [12], and cowpea (*Vigna unguiculata (Linn.) Walp.*) [13]. Moreover, the application of GA_3_ significantly increases the overall height and length of internodes of plants and reduces internode mechanical strength [14,15,16,17,18]. The stimulatory effect of GAs on plant internode length is closely related to the endogenous levels of GAs [19]. After GA_3_ and GA_4_ are applied to pea, it was shown that the contents of GA_1_ and GA_8_ in the stems decrease, while the GA_19_ content increases [20]. In addition, after GA_3_ is applied to soybean, the contents of GA_1_, GA_4_, and jasmonic acid were shown to have increased, while the abscisic acid and salicylic acid contents decreased [21]. Chen et al. [18] reported that the application of GA_3_ reduced the expression of *GA2ox1* in soybean. As shown in a study by Bawa et al. [10], application of GA_3_ promoted GA biosynthesis in soybean, leading to increased GA levels in the plants. Sauter et al. [22] reported that GA_3_ treatment promotes stem elongation by altering the orientation of cellulose microfibrils and that GAs also promote xylem differentiation, regulate cambial cell division, and induce the formation of secondary xylem fibers [23,24,25]. GA_3_ treatment of *Pseudostellaria heterophylla* significantly increased the xylem radius and the number of xylem cell layers in the plants, but it had no significant effect on the width of the secondary phloem, cell layers, or vascular cambium [26]. GA_3_ treatment of *Arabidopsis* also resulted in a significantly increased proportion of xylem [27].

GA_3_ application is an effective way to alter plant height. Most of the current research reports on the application of GA_3_ involve the spraying of leaves or soaking of seeds. To investigate the mechanism of soybean internode elongation in response to GA_3_, we chose Heinong 48 (HN48; a high-stalk cultivar) and Henong 60 (HN60; a dwarf cultivar) [28], two soybean cultivars whose morphologies significantly differ, as experimental materials. After applying GA_3_ to these two soybean cultivars, we examined the effects of GA_3_ on both the soybean internode elongation and the anatomical structure of stem cells and elucidated the pathway through which GA_3_ was transported in soybean stems via the girding method to provide a basis for understanding the mechanism through which GA_3_ regulates soybean internode elongation.

## 2. Results

### 2.1. Effect of GA_3_ Application on Soybean Internode Elongation

As shown in Table 1, the growth patterns of the two soybean cultivars were similar. The first and second internodes of plants treated with V1-GA_3_ were the longest, while the results of the V3-GA_3_ treatment and the control (CK) treatment were not significantly different. In terms of the lengths of the third, fourth, and fifth internodes in response to the different treatments, the patterns were similar; i.e., V1-GA_3_ > V3-GA_3_ > CK, and the difference between treatments was significant. The changes in GA_3_ content in internodes 1–4 were consistent among the treatments, each exhibiting a pattern of V3-GA_3_ > V1-GA_3_ > CK, with a significant difference between treatments. At the vegetative 1 (V1) and V3 stages, the application of GA_3_ to soybean stems increased the internode length and GA_3_ content. However, the V3-GA_3_ treatment was not significant effect on the length of the first and second internodes. Taken together, the results showed that exogenous application GA_3_ to soybean stems could affect the acropetal and basipetal content of GA_3_ in the stems.

The changes in internode length and stem GA_3_ content after GA_3_ was applied to the leaves resulted in patterns that were similar between the two cultivars (Table 2). The lengths of the second and third internodes of the CK plants were not significantly different from those of the Leaf-GA_3_ plants, while the lengths of the fourth, fifth, sixth, and seventh internodes of the Leaf-GA_3_ plants were significantly greater than those of the CK plants. The stem GA_3_ content in plants in the leaf GA_3_ treatment was significantly higher than that in the plants in the CK treatment. On the basis of these results, the application of GA_3_ to leaves promoted stem elongation and increased the stem GA_3_ content but had little effect on the length of the first and second internodes.

As shown in Table 3, the changes in internode length and stem GA_3_ content after GA_3_ was applied to the roots resulted in patterns that were similar between the two cultivars. The lengths of the first and second internodes of plants in the Root-GA_3_ treatment were not significantly different from those of the plants in the CK treatment, but the lengths of the third, fourth, and fifth internodes were significantly greater than those of the plants in the CK treatment. The stem GA_3_ content in the plants whose roots were treated with GA_3_ was significantly higher than that in the CK plants. Taken together, these results indicate that the application of GA_3_ to roots promoted internode elongation and increased the stem GA_3_ content. Similarly, the data in Table 1, Table 2 and Table 3 indicated that the application of GA_3_ to the stems, leaves, and roots of soybean plants increased the internode length and stem GA_3_ content. Notably, although both the V1-GA_3_ and the V3-GA_3_ treatments increased the GA_3_ content in the first internode, the V3-GA_3_ treatment did not significantly increase the length of the first internode, which may be related to the growth period of the internode.

**Figure 1:**
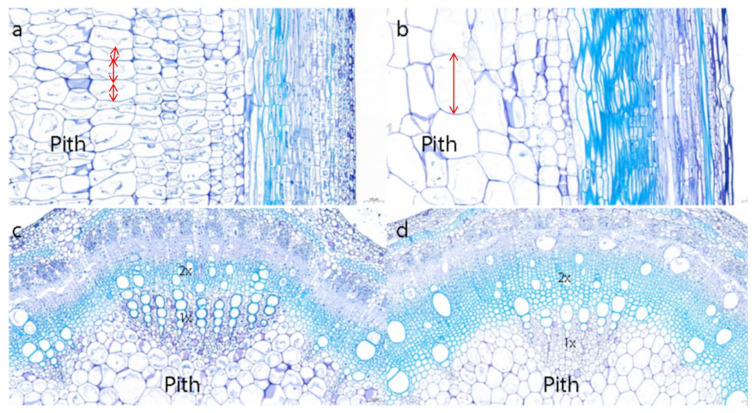
Anatomical structure of soybean internodes (100×). (**a**,**b**) Longitudinal section images of the 2nd internode of CK and V1-GA_3_-treated plants. (**c**,**d**) Cross-sectional images of the 2nd internode of CK and V1-GA_3_-treated plants. 1×, primary xylem; 2×, secondary xylem.

Cross-sectional and vertical sectional images of the soybean internodes of CK plants and V1-GA_3_-treated plants are shown in Figure 1. After GA_3_ was applied, the pith cells, xylem cells, and cortex cells of soybean stems exhibited significant longitudinal elongation. The horizontal length of individual pith cells did not change substantially, while the vertical length of the cells increased by three times compared with that in the CK plants. A comparison of the xylem area in the plants in the two treatment groups showed that, after GA_3_ treatment, the area of secondary xylem in the internodes in the treated plants was larger than that in the CK plants, while the area of primary xylem in the internodes in the treated plants was smaller than that in the CK plants. Therefore, after GA_3_ application, the increase in internode length was consistent with the increase in cell length in the stems, and xylem differentiation in the stems also increased.

### 2.2. Inhibitory Effects of Uniconazole on GA_3_

Table 4 shows the changes in internode length and GA_3_ content after treatment of soybean stems with GA_3_ or uniconazole (S_3307_). The internode lengths and GA_3_ content differed significantly among the four treatments. The lengths of the first and second internodes of the plants in GA_3_+S_3307_ and GA_3_ treatments differed non-significantly but were significantly greater than those of the plants in CK and S_3307_ treatments. Changes in the lengths of the third, fourth and fifth internodes yielded similar patterns: the length of the internodes of plants in the GA_3_ treatment was significantly greater than that in the other three treatments. The length of the internodes of plants in the GA_3_+S_3307_ treatment was greater than that of the plants in the CK and S_3307_ treatments. Moreover, the length of the internodes of plants in the S_3307_ treatment was the shortest, and the fifth internode did not grow at all. The stem GA_3_ content in the plants in the four treatments decreased in the order of GA_3_ > GA_3_+S_3307_ > CK > S_3307_; the difference between the treatments was significant. On the basis of these results, uniconazole reduced the stem GA_3_ content and inhibited the promoting effect of GA_3_ application on internode elongation.

### 2.3. Effects of the Apical Meristem on Soybean Internode Elongation

Table 5 shows the changes in the internode length and stem GA_3_ content after GA_3_ was applied to soybean plants whose apical meristem had been removed. A comparison of the lengths of different internodes indicated that the three treatments did cause significant differences in the length of the second internode and revealed the same pattern (GA_3_ > CK > DW) in terms of the length of the third, fourth, and fifth internodes in the two soybean cultivars, with significant differences occurring between treatments. In HN48, the stem GA_3_ content at the third internode in the GA_3_ treatment was not significantly different from that in the CK treatment. However, for HN60, the difference was significant. In both cultivars, the stem GA_3_ content at the third internode in the DW treatment was significantly lower than that in the other two treatments. The stem GA_3_ content at the fourth and fifth internodes of plants showed the same pattern—GA_3_ > CK > DW. Thus, the apical meristem of soybean plants plays an important role in controlling internode elongation, and the application of GA_3_ restores and increases the GA_3_ level in the stems, promoting internode elongation while substituting for the activity of the apical meristem.

### 2.4. Transport of GA_3_ in Soybean Stem

**Figure 2:**
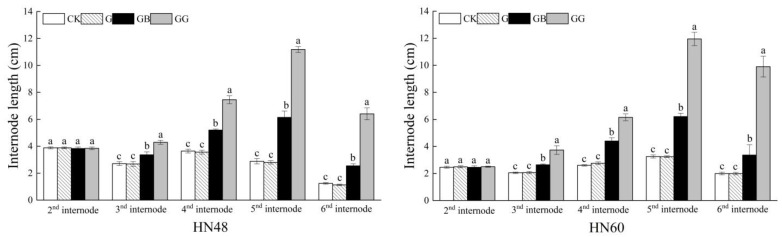
Effects of GA_3_ application on soybean internode length after girding. The values are presented as the means ± standard errors (*n* = 8). The different letters indicate that the differences between treatments reached a significance level of 5%; the treatments are compared longitudinally. CK: control; G: with girding and water application to the incision (water was applied through absorbent cotton at the girding site); GB: with girding and GA_3_ application to the area below the incision at the 2nd internode (water was applied through absorbent cotton at the girding site); GG: with girding and GA_3_ application to the incision (GA_3_ was applied through absorbent cotton at the girding site).

Figure 2 shows the changes in internode length after GA_3_ was applied to different parts of soybean plants after girding. The two cultivars showed the same pattern. The length of the second internode did not differ significantly between the treatments. However, the lengths of the third, fourth, fifth, and sixth internodes of plants in the GG treatment were significantly greater than those in the other three treatments. The lengths of the third, fourth, fifth, or sixth internodes of the plants in the GB treatment were significantly greater than those of the plants in the G or CK treatment, while the length of the internodes of plants in the CK and G treatments did not significantly differ.

**Figure 3:**
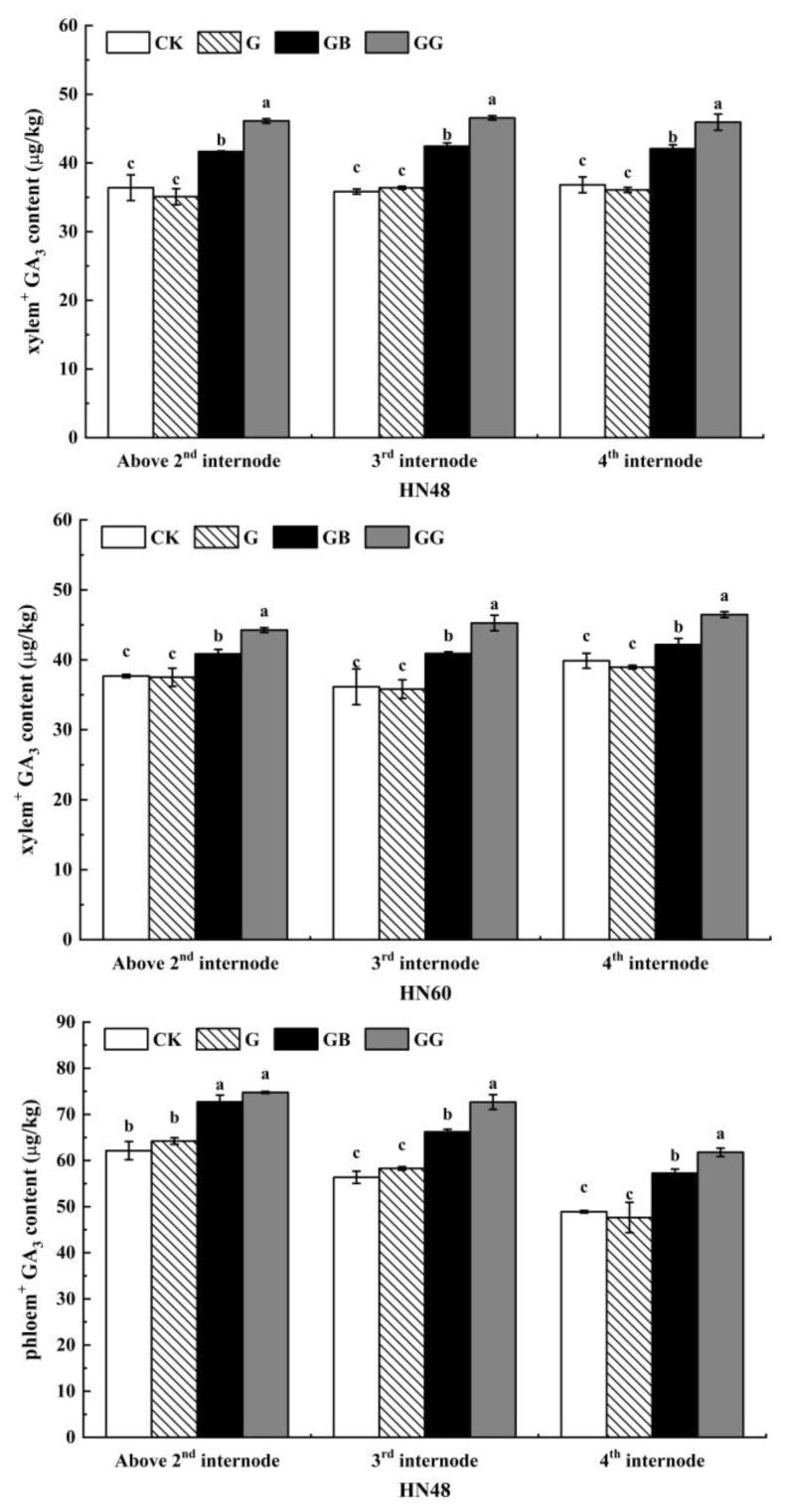

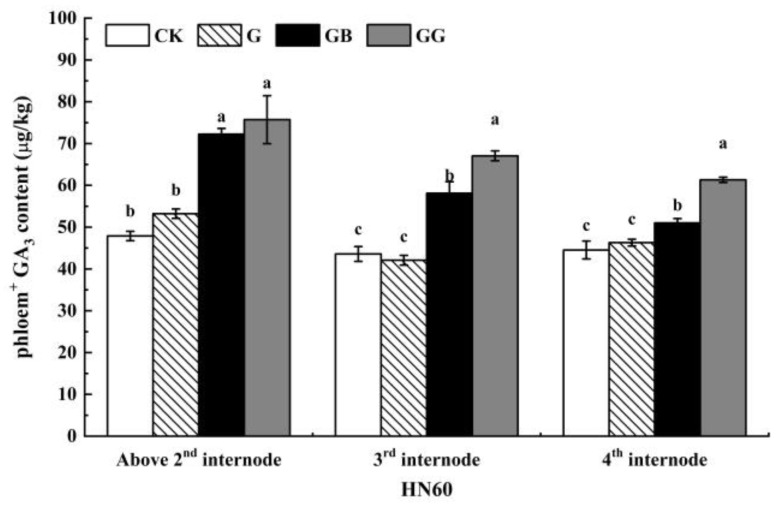
Effects of GA_3_ application on the GA_3_ content in soybean internodes after the girding. The values are presented as the means ± standard errors (*n* = 8). The different letters indicate that the differences between treatments reached a significance level of 5%; the treatments are compared longitudinally. CK: control; G: with girding and water application to the incision (distilled water was applied through absorbent cotton at the girding site); GB: with girding and GA_3_ application to the region below the incision within the 2nd internode (water was applied through absorbent cotton at the girding site); GG: with girding and GA_3_ application to the incision (GA_3_ was applied through absorbent cotton at the girding site).

Figure 3 shows the GA_3_ contents in the xylem^+^ and phloem^+^ of soybean stems. The GA_3_ content in the xylem^+^ at each internode exhibited the same pattern: the GA_3_ content in the plants in the GG treatment was significantly higher than that in the plants in the other three treatment groups; that in the GB treatment group was significantly higher than that in the G and CK treatments; and that in the CK and G treatments did not significantly differ. On the basis of these results, the girding treatment at the second internode did not affect the xylem^+^ GA_3_ content in the soybean plant stems, while the application of GA_3_ at or below the incision increased the xylem^+^ GA_3_ content in the internodes above the second internode, suggesting that the acropetal transport of GA_3_ in soybean plants occurs mainly through the xylem.

A comparison of the GA_3_ content in the phloem^+^ of plants among the four treatments showed a higher phloem^+^ GA_3_ content in the upper part of the second internode of the plants in the GG treatment compared with the GB treatment, but the results of the two treatment groups did not differ significantly. Compared with those in the G and CK treatments, the plants in the GG and GB treatments presented significantly higher phloem^+^ GA_3_ contents, while the content in the plants in the G treatment and CK treatment did not differ significantly. In terms of the phloem^+^ GA_3_ content in the third and fourth internodes of plants, compared with the GB, G, and CK treatments, the GG treatment resulted in significantly higher levels, and compared with the G and CK treatments, the GB treatment resulted in significantly higher levels; however, the contents did not differ significantly between the G treatment and the CK treatment. Taken together, these results showed that the application of GA_3_ to the xylem and stem cortex increased the GA_3_ content in the phloem^+^ of the internodes above the girding site, indicating that GA_3_ in the stem was transported in both directions through the xylem and phloem. Combining the results shown in Figure 2 and Figure 3, we found that the pattern of changes in internode length was essentially consistent with the pattern of changes in GA_3_ content, indicating that the GA_3_ content in the stem is an important factor affecting stem elongation.

## 3. Discussion

GA_1_, GA_3_, GA_4,_ and GA_7_ are the main biologically active forms of GAs and play key roles in plant stem elongation [29,30,31]. The stem elongation of pea [32] and cabbage [33] induced by a low red: far-red (R:FR) ratio is related to increased GA_1_ and GA_4_ levels, and GA_1_ application to pea seedlings has been shown to promote stem elongation [34]. Bensen et al. [35] revealed a positive correlation between soybean hypocotyl growth rate and GA_1_ levels. Liu et al. [36] reported that the increased GA_4_ level observed in intercropped soybean plants was the main reason for the elongated stems of those plants. Zhang et al. [9] applied shade to soybean plants and found that shade promoted internode elongation and increased the stem GA_3_ content. After applying GA_3_ to soybean plants, Hamayun et al. [21] observed increased GA_1_ and GA_4_ levels in the plants along with a significant increase in plant height. Application of GA_3_ restored the plant height of transgenic soybean [37] and rice [38] with a dwarfing phenotype. Moreover, in the present study, the application of GA_3_ to the stems, leaves, and roots of the two soybean cultivars increased the internode length and stem GA_3_ level, which is consistent with the results reported by Zhang et al. [9], indicating that soybean internode elongation is closely correlated with stem GA_3_ content. It was also shown that the application of GA_3_ to stems, leaves, and roots could affect internode elongation. However, it still needs further validation whether exogenous gibberellin can increase the content of GA_3_ in stems by entering different parts of plants or stimulate the synthesis of more gibberellin in the site of synthesis. In addition, Zhang et al. [26] found that the application of GA_3_ to *Pseudostellaria heterophylla* promoted elongation of the stems, while the application of paclobutrazol did not alter the promoting effect of GA_3_. In the present study, we applied uniconazole (S_3307_) to the third internode, while GA_3_ was applied to the second internode of soybean plants in the V2 stage and found that S_3307_ reduced the stem GA_3_ content and inhibited the promotion of internode elongation by GA_3_, which further verifies that the GA_3_ level in the stem is an important factor affecting soybean internode elongation.

The application of GA_3_ to cucumber seedlings was shown to significantly increase the elasticity of the walls of stem cells and promote the elongation of hypocotyls [39]. Moreover, applying GA_3_ to wheat plants at the jointing stage increased the rate of stem elongation but did not affect the height of mature plants [40]. Soybean internode elongation follows the co-elongation rule of three adjacent internodes [9]; the second internode is in the fast elongation phase in the V1 stage but in the slow elongation phase in the V3 stage. In this study, we applied GA_3_ to soybean plants in the V1 and V3 stages separately and found that the effect of GA_3_ application on promoting internode elongation in the V1 stage was significantly stronger than the effect of that in the V3 stage, indicating that the effects of GA_3_ are affected by the growth state of the internode and that the sensitivity of soybean stems to exogenous gibberellin was different in different growth periods. Notably, the application of GA_3_ to either the leaf or the root increased the content of GA_3_ in the stems and promoted elongation of the internodes that were growing. However, for internodes that stopped elongating, GA_3_ application increased the stem GA_3_ level but not the length of the internodes.

GAs are closely related to cell division and expansion [3]. The application of GA_3_ promotes the expression of genes related to cell elongation, expansion, and division in tomato [4]. GA_3_ application significantly promoted the elongation of the root, stem, and leaf cells of *Spondias tuberosa* plants [41]. Eriksson et al. [42] indicated that elevated GA levels increase the number of internode cells in poplar (*Populus*) trees but do not affect the length of epidermal cells or pith cells. In the present study, the application of GA_3_ also increased the longitudinal length of pith, xylem, and cortex cells in soybean stems; however, the width of these cells did not change significantly, indicating that GA_3_ increased the length of the internodes by promoting the longitudinal elongation of internode cells, which is inconsistent with the results reported by Eriksson et al. [42]. We speculated that the stem elongation and growth patterns of different plants were different, which led to the differences in cell shape changes. After GA_3_ was applied, the xylem radius and the number of xylem cell layers of *Pseudostellaria heterophylla* plants increased significantly, but the width of the secondary phloem, the number of secondary phloem cell layers, and the width of the vascular cambium did not significantly change [26]. The application of GA_3_ to different transgenic *Arabidopsis* plants significantly increased the proportion of xylem area relative to the total area [27]. In the present study, we observed that the application of GA_3_ decreased the proportion of pith in the cross-section of soybean stems and the area of primary xylem while increasing the area of the secondary xylem. How exogenous gibberellin regulated internode elongation through cell changes is not yet clear, which also provides a new direction for future research.

GAs are synthesized mainly in the apical parts of higher plants [43]. In rice, young tissues such as flowers and buds are important sites for the synthesis of GAs [44,45], while in pea, GAs are synthesized mainly in immature seeds, young roots, and apical buds, with higher levels detected in actively growing organs, such as unexpanded leaves and elongating internodes [46]. When the tops of sunflower plants were removed, elongation of the lower stems was inhibited, and the inhibition was eliminated by supplementing the plant with 1 mg g^−1^ GA_3_ in agar; elongation of the stem beyond that of the normal growing plant was achieved after GA_3_ supplementation [47]. In the present study, we observed that after the apical meristem was removed from soybean plants, the stem GA_3_ content decreased, and internode elongation was inhibited. However, the application of GA_3_ at the apical meristem removal site restored or even promoted internode elongation while increasing the GA_3_ content in the stems, indicating that the apical meristem is an important site of GA_3_ synthesis in the stems of soybean plants and regulates internode elongation.

GAs may be transported long distances in plants and can act as components of signal transduction [31]; both involve transport through the xylem and the phloem [48]. Ragni et al. [27] reported that GAs were transported basipetally from the apical meristem but also acropetally after they were synthesized in the roots of *Arabidopsis* and that GAs flowed laterally between the phloem and xylem. However, Mauriat et al. [49] argued that lateral flow occurred in only one direction—from the phloem to the xylem. In this study, we applied GA_3_ to the stems of soybean plants with girded stems below the incisions and found that the GA_3_ content increased in both the xylem^+^ and the phloem^+^ of the internodes above the girding incision. We inferred that the GA_3_ absorbed through the cortex is transported to the phloem through the xylem and then acropetally through the xylem. After GA_3_ was applied at the incision, the increases in the GA_3_ content in the phloem^+^ and xylem^+^ of the internodes above the incision were more profound, indicating that, in soybean plants, GA_3_ is transported acropetally through the xylem and laterally between the xylem and phloem.

Lodging was the most important factor that affected soybean yield from a practical point of view, and gibberellin was the main reason that caused stem overgrowth. We believe that the data presented in this study, which provides a theoretical basis for regulating the growth of soybean stem, changing plant height, and alleviating lodging under dense planting conditions in the future.

## 4. Materials and Methods

### 4.1. Materials

A pot experiment was performed from 2019 to 2020 in the experimental field of Northeast Agricultural University, Harbin city, Heilongjiang Province, China (126°36′ E, 45°75′ N). The pots used in this study had an upper diameter of 33.5 cm, a bottom diameter of 24.5 cm, and a depth of 27.5 cm and contained 14 kg of air-dried soil that had been supplemented with 1.50 g of diammonium hydrogen phosphate (N: 18%, P_2_ O_5_: 46%) and 0.75 g of potassium sulfate (K_2_ O: 50%). In each pot, three seedlings (spaced 10 cm apart) were retained. The soil was black soil taken from a corn field and presented the following soil fertility parameters in 2019 and 2020: organic matter, 31.9 g kg^−1^ and 32.7 g kg^−1^, respectively; available N, 63.0 mg kg^−1^ and 50.6 mg/kg, respectively; available P, 75.1 mg kg^−1^ and 53.2 mg kg^−1^, respectively; and available K, 299.0 mg kg^−1^ and 247.0 mg kg^−1^, respectively.

The soybean cultivars used included HN48, which grows to approximately 90 cm, and HN60, which grows to approximately 50 cm [28].

Internode position definition: The first internode extends from the cotyledon mark to the pair of primary leaves, the second internode is located from the pair of primary leaves to the first trifoliolate leaf, the third node is located from the first trifoliolate leaf to the second trifoliolate leaf, and so on [9].

### 4.2. GA_3_ Application

The experiment was conducted in 2019. GA_3_ was applied to soybean stems, leaves, or roots, and GA_3_-inhibiting uniconazole was applied.

GA_3_ application to soybean stems: GA_3_ solution at a concentration of 0.1 g L^−1^ was applied to the second internode in the V1 stage and V3 stage (denoted V1-GA_3_ and V3-GA_3_, respectively) for three consecutive days, with normally growing plants serving as CKs. Samples were collected from plants in the V5 stage to measure the internode length and stem GA_3_ content. Samples were taken from the middle section of the second internode of the V1-GA_3_-treated plants and CK plants, from which paraffin sections were prepared.

GA_3_ application to soybean leaves: Beginning in the V4 stage, 0.1 g L^−1^ GA_3_ solution was applied to the first and second trifoliolate leaves daily, the plants of which were denoted as Leaf-GA_3_ plants, with the normal plants serving as CKs. When the seventh trifoliolate leaf that emerged from the CK plants, samples were collected to measure the internode length and stem GA_3_ content.

GA_3_ application to soybean roots: Sand culture was adopted in accordance with the nutrient solution recipe and cultivation method described by Lyu et al. [50]. Starting from the V4 stage, the roots of soybean plants were continuously watered with a nutrient solution supplemented with GA_3_ (0.1 g L^−1^), the plants of which were denoted as Root-GA_3_ plants, with the normal plants serving as CKs. When the seventh trifoliolate leaf emerged from the CK plants, samples were collected to measure the internode length and stem GA_3_ content.

Inhibitory effects of uniconazole on GA_3_: Starting from the V2 phase, four treatments were established, GA_3_ (GA_3_ applied to the second internode), S_3307_ (uniconazole applied to the second internode), GA_3_-S_3307_ (GA_3_ applied to the second internode and uniconazole applied to the third internode), and CK. Uniconazole was applied until the seventh trifoliolate leaf emerged from the plants in the CK treatment group, after which samples were collected to measure the internode length and stem GA_3_ content.

### 4.3. Role of the Apical Meristem in Soybean Internode Elongation

The experiment was conducted in 2019. At the V4 stage, the apical meristem was removed, and the wound was then wrapped with 0.2 g of absorbent cotton, to which a 0.1 g L^−1^ GA_3_ solution (GA_3_) or distilled water (DW) was applied daily. Plants from which the apical meristem was not removed were designated the CK group. When the seventh trifoliolate leaf emerged from the CK treatment group, samples were collected to measure the internode length and stem GA_3_ content.

### 4.4. GA_3_ Transport Experiment in Soybean Stem

The experiment was conducted in 2020. At the V3 stage, the cortex with phloem (width of 5.0 mm) in the middle of the second node of soybean stems was removed by a sterilized blade, and the wound was then wrapped with absorbent cotton to prevent dehydration. Four treatments were established: GB (0.1 g L^−1^ GA_3_ solution applied to an area below the incision), GG (0.1 g L^−^^1^ GA_3_ solution applied to the incision area), G (DW applied to the incision area), and a CK (intact plants). Twelve plants per treatment were established, and samples were collected at the V5 stage. The length of each internode was subsequently measured. The internodes were separated into phloem^+^ (which included the epidermis, cortex, and phloem) and xylem^+^ (which included the xylem and pith), after which their GA_3_ contents were determined.

### 4.5. Measurements and Paraffin Section Preparation

Internode length measurement: The length of each internode of soybean plants was measured with a ruler.

Preparation of paraffin sections: Samples were fixed with a mixture of formaldehyde, acetic acid, and ethanol (FAA) for 48 h [51] and then transferred to ethanol for stepwise dehydration, after which paraffin sections were prepared. The sections were stained with toluidine blue and observed under an upright optical microscope (Nikon Eclipse E100, Tokyo, Japan), and images were taken with an imaging system (Nikon DS-U3, Tokyo, Japan).

Stem GA_3_ content determination: An enzyme-linked immunosorbent assay (ELISA) was used to determine the GA_3_ content. After the samples were cut, 0.1 g of the freeze-dried sample tissue was accurately weighed, and PBS (pH = 7.4) solution was added for even grinding. The samples were then maintained at 2–8 °C after melting, after which they were centrifuged at 4000 r min^−1^ for 15 min. The supernatant was then collected and added to an ELISA plate. After adding the enzymes, incubating, washing, coloring, and stopping the reaction, with blank wells serving controls, we determined the absorbance at 450 nm. Stop solution was then added, and within 15 min, the concentration of GA_3_ was calculated. Each sample was tested eight times.

### 4.6. Statistical Analysis

Analysis of variance (ANOVA) in SPSS software v.17.0 (SPSS, Chicago, IL, USA) was used to analyze the different treatments; each treatment was repeated eight times. The means were compared via one-way ANOVA followed by least-significant difference tests at the *p* ≤ 0.05 level. The figures were generated using Origin 9.0 (OriginLab, Northampton, MA, USA) and Microsoft Publisher software.

## 5. Conclusions

Application of GA_3_ to the stems, leaves, and roots of soybean plants increased the internode length and stem GA_3_ content. The application of GA_3_ decreased the proportion of pith in soybean stems and the primary xylem area but increased the secondary xylem area. The apical meristem is an important site of GA_3_ synthesis in soybean stems and regulates stem elongation. GA_3_ was transported acropetally through the xylem and laterally between the xylem and phloem in the soybean stems. We conclude that the GA_3_ level in stems is an important factor affecting internode elongation.

## Figures and Tables

**Table 1:** Effects of GA_3_ application to soybean stems on internode elongation and stem GA_3_ content.

Cultivar	Node Position	Internode Length (cm)			GA_3_ (μg kg^−1^)		
		CK	V_1_-GA_3_	V_3_-GA_3_	CK	V_1_-GA_3_	V_3_-GA_3_
HN48	1	7.20 ± 0.12 b	7.90 ± 0.09 a	7.13 ± 0.08 b	31.25 ± 1.27 c	34.65 ± 0.35 b	37.36 ± 0.42 a
	2	3.62 ± 0.03 b	9.66 ± 0.57 a	3.66 ± 0.03 b	29.26 ± 0.85 c	35.07 ± 0.61 b	40.33 ± 1.71 a
	3	2.64 ± 0.09 c	13.46 ± 0.29 a	4.56 ± 0.44 b	27.54 ± 1.24 c	33.75 ± 0.30 b	37.76 ± 0.14 a
	4	4.48 ± 0.11 c	19.52 ± 0.21 a	8.40 ± 0.29 b	24.31 ± 0.55 c	28.44 ± 0.41 b	33.57 ± 0.39 a
	5	5.18 ± 0.08 c	10.24 ± 0.20 a	6.55 ± 0.12 b	-	-	-
HN60	1	5.15 ± 0.07 b	6.73 ± 0.12 a	5.23 ± 0.10 b	30.22 ± 0.75 c	33.51 ± 0.18 b	35.46 ± 0.49 a
	2	2.66 ± 0.05 b	8.08 ± 0.18 a	2.78 ± 0.12 b	27.34 ± 0.39 c	34.18 ± 0.47 b	38.18 ± 0.40 a
	3	2.22 ± 0.05 c	13.18 ± 0.21 a	3.18 ± 0.02 b	26.42 ± 0.47 c	28.78 ± 0.20 b	30.10 ± 0.39 a
	4	2.70 ± 0.04 c	13.56 ± 0.31 a	5.72 ± 0.14 b	23.20 ± 0.50 c	27.50 ± 0.78 b	31.89 ± 0.41 a
	5	3.20 ± 0.12 c	9.18 ± 0.13 a	5.70 ± 0.22 b	-	-	-

The values are presented as the means ± standard errors (*n* = 8). The different letters indicate that the differences between treatments reached a significance level of 5%; the treatments are compared horizontally. V1-GA_3_: GA_3_ solution (0.1 g L^−1^) was applied to the 2nd internode in the V1 phase; V3-GA_3_: GA_3_ solution (0.1 g L^−1^) was applied to the 2nd internode in the V3 phase; CK: control.

**Table 2:** Effects of GA_3_ application to soybean leaves on internode elongation and stem GA_3_ content.

Cultivar	Internode Position	Internode Length (cm)		GA_3_ (μg kg^−1^)	
		CK	Leaf-GA_3_	CK	Leaf-GA_3_
HN48	2	3.58 ± 0.07 a	3.56 ± 0.07 a	30.10 ± 0.75 b	33.59 ± 0.93 a
	3	3.22 ± 0.02 a	3.20 ± 0.05 a	26.77 ± 0.76 b	31.53 ± 1.08 a
	4	3.30 ± 0.07 b	3.80 ± 0.12 a	25.22 ± 0.93 b	30.26 ± 1.71 a
	5	3.81 ± 0.18 b	6.30 ± 0.31 a	24.47 ± 0.46 b	26.86 ± 1.02 a
	6	4.28 ± 0.02 b	12.48 ± 0.11 a	23.83 ± 0.32 b	28.59 ± 0.94 a
	7	4.98 ± 0.17 b	11.90 ± 0.19 a	21.98 ± 0.30 b	25.31 ± 0.10 a
HN60	2	2.30 ± 0.00 a	2.22 ± 0.06 a	26.50 ± 2.29 b	30.40 ± 1.94 a
	3	2.00 ± 0.00 a	2.14 ± 0.05 a	23.77 ± 0.56 b	28.34 ± 1.51 a
	4	1.90 ± 0.05 b	2.35 ± 0.06 a	24.39 ± 0.61 b	29.81 ± 0.24 a
	5	2.18 ± 0.04 b	3.29 ± 0.04 a	23.48 ± 1.22 b	25.96 ± 0.30 a
	6	3.40 ± 0.06 b	6.36 ± 0.13 a	22.72 ± 0.83 b	24.79 ± 1.13 a
	7	4.18 ± 0.17 b	8.32 ± 0.20 a	21.87 ± 0.63 b	23.88 ± 0.75 a

The values are presented as the means ± standard errors (*n* = 8). The different letters indicate that the differences between treatments reached a significance level of 5%; the treatments are compared horizontally. CK: control; Leaf-GA_3_: GA_3_ solution (0.1 g L^−^^1^) was applied daily at the beginning of the V4 stage to the 1st and 2nd trifoliolate leaves of soybean plants.

**Table 3:** Effects of GA_3_ application to the roots of soybean plants on internode length and stem GA_3_ content.

Cultivar	Internode Position	Internode Length (cm)		GA_3_ (μg kg^−^^1^)	
		CK	Root-GA_3_	CK	Root-GA_3_
HN48	1	6.25 ± 0.07 a	6.27 ± 0.06 a	19.37 ± 0.16 b	21.09 ± 0.20 a
	2	4.28 ± 0.11 a	4.30 ± 0.24 a	14.93 ± 0.11 b	16.45 ± 0.15 a
	3	4.52 ± 0.06 b	5.35 ± 0.07 a	14.85 ± 0.21 b	18.55 ± 0.09 a
	4	6.12 ± 0.07 b	9.00 ± 0.31 a	15.78 ± 0.14 b	18.91 ± 0.12 a
	5	5.20 ± 0.13 b	9.38 ± 0.32 a	12.62 ± 0.24 b	17.60 ± 0.31 a
HN60	1	4.12 ± 0.04 a	4.14 ± 0.06 a	15.04 ± 0.29 b	16.83 ± 0.21 a
	2	2.48 ± 0.05 a	2.45 ± 0.05 a	14.14 ± 0.09 b	15.97 ± 0.06 a
	3	3.00 ± 0.08 b	3.66 ± 0.05 a	13.46 ± 0.05 b	18.13 ± 0.42 a
	4	3.90 ± 0.08 b	5.78 ± 0.13 a	14.31 ± 0.10 b	17.78 ± 0.17 a
	5	3.70 ± 0.05 b	7.33 ± 0.20 a	12.27 ± 0.18 b	16.11 ± 0.14 a

The values are presented as the means ± standard errors (*n* = 8). The different letters indicate that the differences between treatments reached a significance level of 5%; the treatments are compared horizontally. CK: control; Root-GA_3_: GA_3_ solution (0.1 g L^−1^) was applied to the roots of soybean plants.

**Table 4:** Effects of GA_3_ and uniconazole application on soybean internode length and stem GA_3._

	Internode Position/Treatment	CK	S_3307_	GA_3_	GA_3 +_ S_3307_
Internode length (cm)	1	5.66 ± 0.13 b	4.60 ± 0.10 c	6.27 ± 0.14 a	6.20 ± 0.12 a
	2	3.68 ± 0.06 b	2.71 ± 0.12 c	7.33 ± 0.20 a	7.28 ± 0.14 a
	3	3.54 ± 0.03 c	1.23 ± 0.14 d	16.67 ± 1.24 a	11.54 ± 0.41 b
	4	3.51 ± 0.13 c	0.83 ± 0.01 d	26.53 ± 0.59 a	18.04 ± 0.29 b
	5	0.94 ± 0.05 c	-	9.45 ± 0.31 a	3.04 ± 0.23 b
GA_3_ (μg kg^−1^)	1	29.99 ± 0.17 c	25.15 ± 0.36 d	34.65 ± 0.20 a	31.58 ± 0.42 b
	2	29.44 ± 0.12 c	24.70 ± 0.28 d	37.36 ± 0.24 a	35.37 ± 0.38 b
	3	25.93 ± 0.40 c	21.37 ± 0.52 d	35.07 ± 0.35 a	32.64 ± 0.44 b
	4	23.38 ± 0.14 c	19.57 ± 0.49 d	32.23 ± 0.37 a	30.82 ± 0.23 b

The values are presented as the means ± standard errors (*n* = 8). Different letters indicate that the differences between treatments reached a significance level of 5%; the treatments are compared horizontally. CK: control; S_3307_: S_3307_ was applied to the 2nd internode at the V1 stage; GA_3_: GA_3_ was applied to the 2nd internode at the V1 stage; GA_3_+S_3307_: GA_3_ was applied to the 2nd internode at the V1 stage, after which S_3307_ was applied to the 3rd internode.

**Table 5:** Effects of GA_3_ application on soybean internode length and stem GA_3_ content after apical meristem removal.

Cultivar	Internode Position	Internode Length (cm)			GA_3_ (μg kg^−1^)		
		CK	DW	GA_3_	CK	DW	GA_3_
HN48	2	4.00 ± 0.00 a	3.97 ± 0.01 a	4.00 ± 0.02 a	-	-	-
	3	3.77 ± 0.01 b	3.29 ± 0.03 c	4.06 ± 0.01 a	36.43 ± 1.71 a	29.87 ± 0.60 b	37.54 ± 1.16 a
	4	4.11 ± 0.02 b	2.86 ± 0.05 c	5.09 ± 0.06 a	32.44 ± 0.49 b	28.84 ± 0.41 c	35.69 ± 0.28 a
	5	4.56 ± 0.07 b	2.03 ± 0.04 c	7.98 ± 0.02 a	30.30 ± 0.48 b	25.82 ± 0.75 c	34.81 ± 0.26 a
HN60	2	3.16 ± 0.09 a	3.09 ± 0.06 a	3.11 ± 0.04 a	-	-	-
	3	2.71 ± 0.02 b	2.30 ± 0.06 c	3.06 ± 0.03 a	28.55 ± 0.60 b	23.56 ± 0.64 c	32.00 ± 1.66 a
	4	3.22 ± 0.08 b	2.42 ± 0.02 c	4.26 ± 0.02 a	25.61 ± 1.09 b	22.60 ± 0.66 c	30.32 ± 0.49 a
	5	2.96 ± 0.05 b	1.81 ± 0.01 c	5.23 ± 0.04 a	25.33 ± 0.76 b	21.52 ± 0.29 c	29.73 ± 1.18 a

The values are presented as the means ± standard errors (*n* = 8). The different letters indicate that the differences between treatments reached a significance level of 5%; the treatments are compared horizontally. CK: control; DW: apical meristem removed and distilled water applied; GA_3_: apical meristem removed and GA_3_ applied.

## Data Availability

The authors confirm that all data, tables, and figures in this manuscript are original.
